# Laparoscopic Approach to Inguinal Disruption in Athletes: a Retrospective 13-Year Analysis of 198 Patients in a Single-Surgeon Setting

**DOI:** 10.1186/s40798-019-0201-4

**Published:** 2019-06-24

**Authors:** Guglielmo Niccolò Piozzi, Riccardo Cirelli, Ilaria Salati, Marco Enrico Mario Maino, Ennio Leopaldi, Giovanni Lenna, Franco Combi, Giuseppe Massimiliano Sansonetti

**Affiliations:** 1 General Surgery Residency Program, Università degli Studi di Milano, Via Festa del Perdono, 7, 20122 Milan, Italy; 2 Department of General Surgery, Ospedale S. Carlo Borromeo, Via Pio II, 3, 20153, Milan, Italy; 3 Deparment of General Surgery, Casa di Cura Igea, Via Marcona, 69, 20129 Milan, Italy; 4 Football Medical Staff, Sassuolo Calcio, Sassuolo, Italy

**Keywords:** Inguinal disruption, Sport’s hernia, Sportsman’s groin, Gilmore’s groin, TAPP, Mesh fixation, Surgical glue

## Abstract

**Background:**

Inguinal disruption (ID) is a condition of chronic groin pain affecting mainly athletes. ID cannot be defined as a true hernia. Pathogenesis is multifactorial due to repetitive and excessive forces applied to the inguino-pelvic region. Examination reveals tenderness to palpation of the inguinal region. Differential diagnosis is challenging; imaging is helpful for excluding other pathologies. Surgery is the treatment of choice when conservative treatment fails. Primary aim of the study was to evaluate the time to return to full sport activity after transabdominal preperitoneal patch plasty (TAPP) technique in ID. Secondary aim was to evaluate the postoperative complication rate both in the immediate post-operative time and in 1 year follow-up and to verify the relapse rate after surgery. In this study, we consider time to return to full sport activity as the time needed to return to pre-injury sport activity.

**Results:**

A retrospective study is reported by evaluating 198 cases of ID from a single surgeon experience. All patients failed a previous conservative treatment. All cases were treated with the TAPP approach. Time to return to full sport activity was 4 weeks for 94.4% of patients, with a total of 98.5% of active patients at 9 months. Post-operative inguinal pain was the main complication (9.1%). On 13 years follow-up, we report a recurrence rate of 2.5%.

**Conclusions:**

Current management algorithm for ID, in professional athletes, supports the role of surgery after at least 2 months of conservative treatment. Recently, the role of surgery has been highlighted for a definitive treatment and a faster full recovery to sport activity, especially for elite professional athletes. In our opinion, laparoscopic surgery is the mainstay for non-responsive ID treatment. We present a long-term retrospective evaluation of a wide cohort of professional athletes diagnosed and treated in a systematic way.

## Key Points

- Surgery is the treatment of choice for ID when conservative treatment fails
- Laparoscopic technique offers an advantage over open surgery in terms of faster rehabilitation, earlier return to daily and full sport activities
- TAPP repair should be performed bilaterally in ID in order to have a better outcome

## Background

Inguinal disruption (ID) is a clinical condition of chronic groin pain affecting many athletes, both professional and amateur [1]. In literature, several non-specific terms are reported including sport’s hernia, chronic groin pain, sportsman’s hernia, sportsman’s groin, athletic pubalgia, Gilmore’s groin, ice hockey groin and pubic inguinal pain syndrome (PIPS) [1–5]. The Manchester Consensus Conference of the British Hernia Society (BHS) and the Doha Meeting [3, 4] managed to standardize the terms and features of this syndrome. The BHS agreed on the term “inguinal disruption” for defining this condition [4].

ID is often found in athletes who undertake sports involving kicking and twisting movements while running [4]. ID accounts for 6% of all athletic injuries [6]. ID affects about 0.5–6.2% of all athletes, with major incidence between soccer (males 10–18%; females 2–14%) [3, 7] and hockey players (13–20%) [8]. The median age of athletes ranges between 26 and 28 years [6].

Because of the absence of an abnormal exit of tissue through the abdominal wall in the inguinal region, ID cannot be defined as a “true” hernia [4]. Pathogenesis is multifactorial, and the final outcome is a posterior abdominal wall weakness in the inguinal area with subsequent genesis of abnormal tension in the inguinal canal [4, 9–11]. Therefore, the unbalanced force at the pelvis and the inguinal canal is the specific cause of ID [4]. Weakness is due to repetitive and excessive force applied to the inguinal and pelvic region during inferior limb movements especially of adductors, caudal portion of abdominal wall muscles and hip articulation [4, 9, 12].

Patients report monolateral groin pain at the pubic symphysis radiating through the scrotum, the perineum, the rectus abdominis and the adductor tendon (according to nerve distributions) with associated resistance or pain on ipsilateral hip adduction [9, 12]. Symptoms are worsened by athletic activity such as kicking, cutting or sprinting, especially with acceleration and deceleration, and are alleviated by rest [2, 4, 9]. Examination reveals tenderness to palpation of the inguinal region just above the inguinal ligament and near the pubis, and it is worsened by coughing and by performing Valsalva manoeuvre [9]. A visible or palpable inguinal mass, if present, points to a true hernia and not to ID [3, 4]. Differential diagnosis of inguinal pain is shown in Table 1 [9].

**Table 1:** Differential diagnosis of inguinal pain [9]

Proposed causes of sports hernia	
Conjoined tendon inflammation or tear Inguinal ligament tear External oblique muscle tear Posterior abdominal wall attenuation Superficial inguinal ring dilation	
Inguinal location not related to sports hernia	
Inguinal hernia Nerve compression	
Others	
Pubic instability Osteitis pubis Adductor strain or tear Femoroacetabular impingement Iliopsoas strain or tear Snapping iliopsoas Rectus abdominis strain or tear	

Diagnosis of ID is a clinical challenge, and for this reason, the BHS has proposed diagnostic criteria for sports hernia (Table 2) [4]. It is important to detect possible secondary or tertiary clinical entities associated to ID in order to improve the clinical outcomes (e.g. femoroacetabular impingement) [13]. The first step is to evaluate the bone structures of the pelvis and hip through a radiograph and to perform a detailed differential diagnosis [9]. Magnetic resonance imaging (MRI) is considered the preferred diagnostic imaging for ID, especially for excluding other pathologies [4].

**Table 2:** British Hernia Society Criteria for sports hernia diagnosis [4]

British Hernia Society Criteria for sports hernia diagnosis (> 3 following criteria)	
Pain that is described dull or diffuse that radiates to the medial aspect of the thigh, perineum or contralateral side	
Tenderness to palpation over the pubic tubercle at the insertion of the inguinal ligament	
Tenderness to palpation of the deep inguinal ring	
Tenderness to palpation of the adductor longus tendon	
Tenderness or dilation of the superficial inguinal ring	

The BHS proposed a treatment algorithm (Table 3) [4]. ID must be approached at first with specific sport rehabilitation programs and physiotherapy for 2–6 weeks [4]. If conservative therapies and physiotherapy fails, surgery becomes the treatment of choice. The surgical repair of ID can be performed by either open (OT) or laparoscopic technique (LT; transabdominal preperitoneal patch plasty (TAPP) and total extraperitoneal patch plasty (TEP)) with similar results in the recognition of local anatomy and the release of abnormal tension in the inguinal ligament.

**Table 3:** British Hernia Society suggested management of ID [4]

Time (months)	Discomfort	Treatment
1–2	ID; VAS 0–2 at rest; VAS 6–7 on exercise: no sport activity	Prehabilitation, rest and analgesia
> 2	Ongoing ID—chronic groin pain: failure of rehabilitation	Surgical repair (open/laparoscopic) and postop rehabilitation

Since the LT is associated to a quicker recovery and return to sporting activity, the primary aim of the study was to evaluate the time to return to full sport activity after the TAPP technique in ID. Secondary aim was to evaluate the postoperative complication rate both in the immediate post-operative time and in 1 year follow-up and to verify the relapse rate after surgery. In this study, we consider time to return to full sport activity as the time needed to return to pre-injury sport activity.

## Methods

### Patients’ Characteristics

**Fig. 1:**
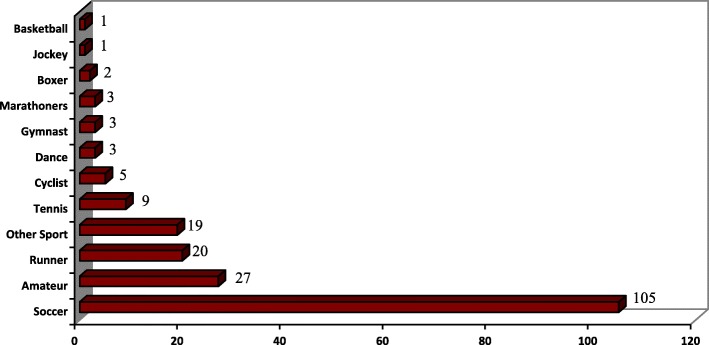
Sport distribution of the patients

A retrospective study was performed evaluating all patients with ID submitted to laparoscopic surgical treatment with the TAPP technique. A total of 198 patients (*n*: male/female 185/13) were retrieved between May 2004 and July 2017. All patients were sportsmen from different levels of competition, both amateurs and professional (e.g. Italian Serie A and B soccer players). All surgical procedures were performed by the same surgeon (Giuseppe Massimiliano Sansonetti) at “Ospedale Bassini” (between 2004 and 2012) and “Casa di Cura Igea” (between 2012 and 2017) in Milan (Italy). All patients were referred to our attention from Professional Sport Medical Services and Physiotherapy Service. One hundred eighty-three patients (92.4%) were submitted, before surgery, to dynamic abdominal wall ultrasound (dUS) evaluation performed by a skilled radiologist. During dUS, a real-time convex anterior bulge and ballooning of the inguinal canal was observed at the superficial inguinal ring. dUS showed, in 175 patients (88.4%), a bilateral or monolateral inguinal bulge with evidence of peritoneal fat ballooning during Valsalva manoeuvre. dUS was negative in 8 cases; further abdominal magnetic resonance imaging (MRI) confirmed an inguinal ballooning in 5 cases, with exclusion of further concurrent skeletal-muscle injuries. A total of 15 patients were not submitted to a preoperative dUS. These cases belong to the first surgical period when treatment was not fully standardized; it is therefore a partial limitation of this study. All patients underwent bilateral TAPP repair. The mean age of the patients was 24 years (range 14–42 years). Male patients were as follows: 105 soccer players (53.0%), 20 runners (10.1%), 7 tennis players (3.5%), 5 cyclists (2.5%), 2 boxers (1.0%), 1 jockey (0.5%), 1 basketball player (0.5%), 19 athletes from other specialties (9.7%) and 25 amateur athletes (12.7%). Female patients were as follows: 3 dancers (1.5%), 3 gymnasts (1.5%), 3 marathoners (1.5%), 2 tennis players (1.0%) and 2 amateur athletes (1.0%) (Fig. 1). All percentages are referred to the study pool (*n* = 198). Data were analysed using the Microsoft Excel for Windows Version 8.0 (Microsoft Corporation, USA).

### Symptoms

The patients’ detected symptoms were as follows: 121 monolateral inguinal pain (61.1%), 53 bilateral inguinal pain (26.8%), 14 pubalgia (7.1%), 5 scrotal and perineal pain (2.5%) and 5 hypogastric pain (2.5%). At the first surgical visit, 143 patients (72.2%) presented ID symptomatology with inguinal bilateral impulse during Valsalva manoeuvre whereas 55 patients (27.8%) presented monolateral inguinal symptoms. In all cases, the symptomatology was referred invalidating.

### Surgical Technique

**Fig. 2:**
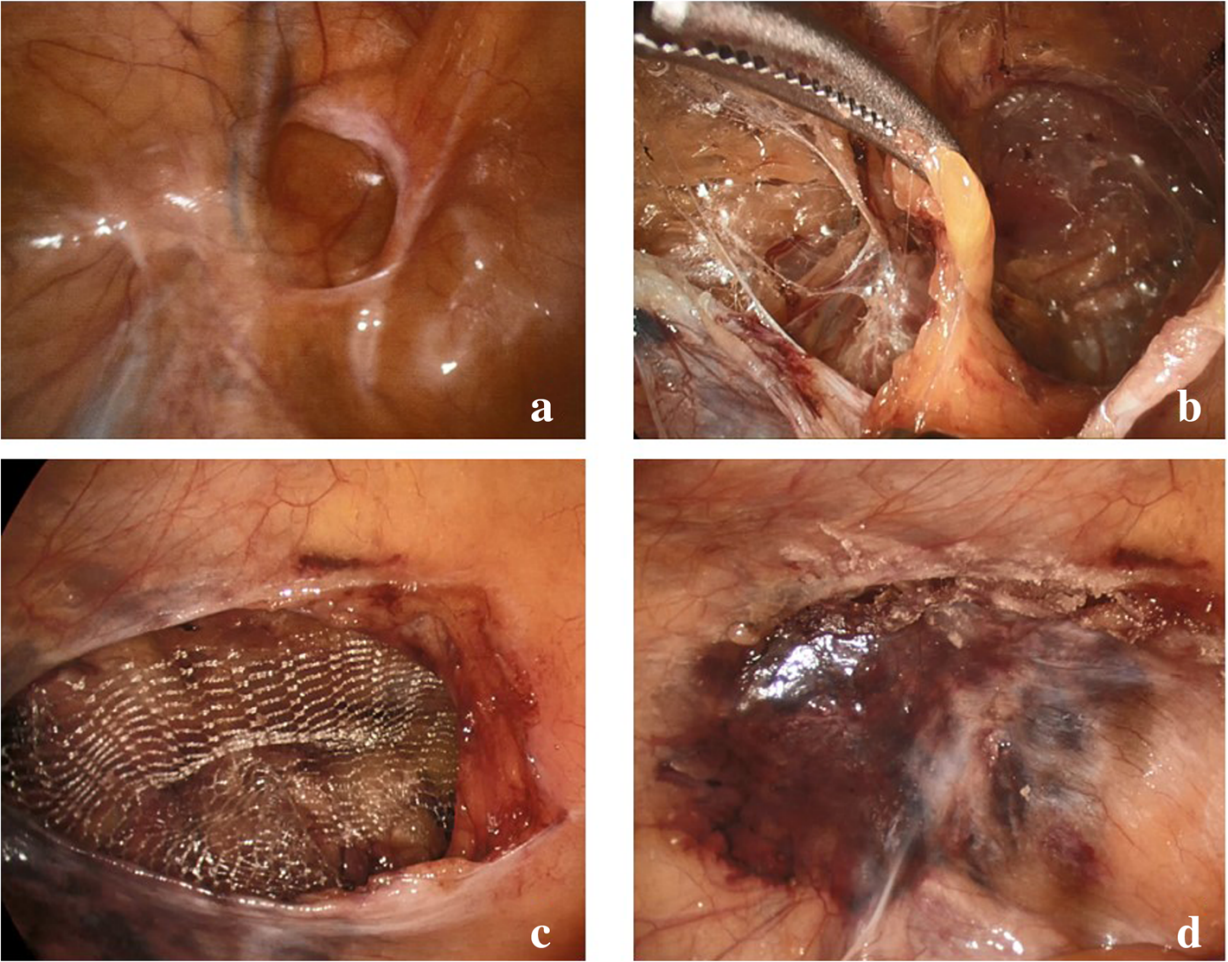
Steps of TAPP technique on ID (left groin): **a** intraoperative visualization of ID, **b** peritoneal fat bulge mobilization and removal, **c** positioning and fixation of the mesh with surgical glue, and **d** closure of the peritoneal incision by peritoneal flaps overlapping and fixation with surgical glue

All patients were treated with the standard TAPP technique under general anaesthesia (Fig. 2). A 14-mmHg pneumoperitoneum was achieved with a Veress technique. Three trocars were used: a 12-mm optical trocar was placed in the supraumbilical position; two 5-mm operating trocars were placed at the intersection of the right and left midclavicular line with the horizontal umbilicus line. The abdominal cavity was explored through a 30° optical camera excluding conditions that may contraindicate the laparoscopic procedure. A bilateral TAPP procedure was performed according to the standard technique paying great attention during dissection to avoid any anatomical injuries in the “pain” and “doom” triangles. The exposed peritoneal fat bulge was freed from the surrounding tissues and was removed. In all patients, a 15 × 10 cm non-absorbable polypropylene monofilament mesh (BULEV B®; weight 48 g/m^2^ ± 10%; mean pore area 1.5 mm^2^; mean pore dimension 2.76 mm; mean thickness 0.56 mm ± 10%) was tailored and positioned bilaterally. The mesh was then fixed by applying n-hexyl/cyanoacrylate glue (IFABOND®; Péters Surgical; Bobigny, France) in order to prevent recurrences and reduce possible stapling complications (lateral cutaneous nerve of the thigh or the femoral branch of the genitofemoral nerve injury) or vascular lesions (“triangle of pain” and “triangle of doom”). The peritoneal incision closure was achieved by overlapping the peritoneal flaps, and fixation was performed, with a tension-free technique, with surgical glue (IFABOND®; Péters Surgical; Bobigny, France). In order to facilitate the peritoneal closure, the pneumoperitoneum was reduced to 12 mmHg.

## Results

All patients underwent conservative treatment with anti-inflammatory drugs (either oral or through local injection), rest and physiotherapy protocols (core stability and abdominal/hip muscles exercises) for at least 6 weeks [14]. However, few patients referred a partial benefit with symptom recurrence after return to sport activities. Following conservative treatment failure, all 198 patients were submitted to bilateral inguinal repair through the TAPP technique. No laparoscopic to open conversion was needed. A bilateral bulge of peritoneal fat in the inguinal area was evidenced in all 198 patients during surgery (intraoperative inguinal finding combinations are described in Table 4).

**Table 4:** Intraoperative inguinal finding combinations

Patients (%)	Intraoperative inguinal finding combinations	
100 (50.5%)	Direct	Direct
35 (17.7%)	Indirect	Indirect
55 (27.8%)	Direct	External oblique
7 (3.5%)	Direct	Internal oblique
1 (0.5%)	Direct	Femoral

Mean operative time was 90 min (range 50–140). Fluid oral intake was administered in post-operative day (POD) 0 with full solid diet on POD 1. Mobilization was performed in POD 0. One hundred ninety-seven patients (99.5%) were discharged in POD 1 with no postoperative complications. One patient’s discharge (0.5%) was delayed to POD 5 following haemorrhage in POD1 with laparoscopic revision and haemostasis of testicular vessels’ bleeding.

One patient (0.5%) was readmitted at POD3 with small bowel occlusion; the patient was submitted to laparoscopic revision with evidence of an internal hernia in the preperitoneal right space due to detachment of the peritoneal flap. The internal hernia was corrected, and the peritoneal flap was closed with surgical glue. Following postoperative progress was uneventful.

The mean return time to routine daily life was 48 h (range 24–120) with postoperative pain being the main delaying factor.

Follow-up regimen was characterized by a clinical evaluation and was scheduled as follows: 7–10 days, 1 month, 3 months and 12 months.

**Fig. 3:**
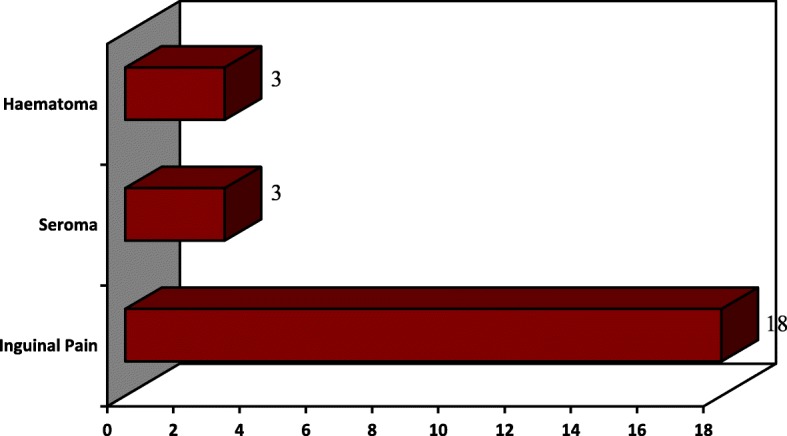
Complications’ distribution

A total of 24 patients (12.1%) referred an early complication at follow-up day 7: 3 patients (1.5%) had a small periumbilical haematoma with spontaneous resolution; 3 patients (1.5%) had a subcutaneous seroma in the inguinal region (1 was treated with needle evacuation while 2 resolved spontaneously); 18 patients (9.1%) had inguinal pain treated with anti-inflammatory and rest (Fig. 3).

**Fig. 4:**
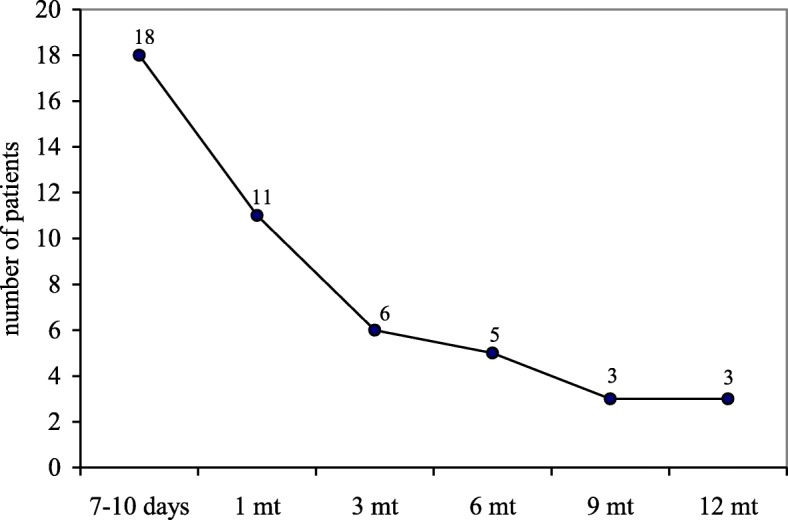
Post-surgical inguinal pain follow-up; mt: months

**Fig. 5:**
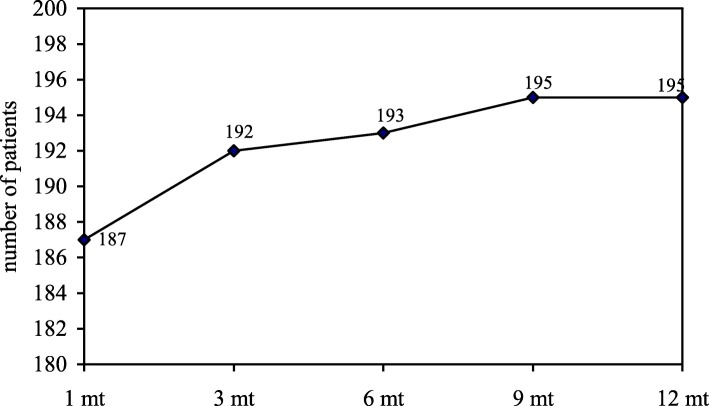
Time to return to sport activity; mt: months

At 1 month follow-up, a total of 11 patients (5.6%) referred persistent inguinal pain partially responsive to analgesics with subsequent delay in returning to sport activity; meanwhile, 187 patients (94.4%) started full physical activity after 4 weeks (range 3–6) (Figs. 4 and 5).

At 3 months, 5 (2.5%) of the 11 aforementioned patients referred symptom benefit after conservative treatment with return to sport activity after 12 weeks (range 10–16). The remaining 6 patients (3.0%) referred inguinal pain not responsive to analgesics with inguinal US negative for hernia recurrence. Those patients were treated conservatively with 3 months follow-up. One patient (0.5%) returned to sport activity after 6 months, 2 patients (1.0%) after 9 months while the remaining 3 patients (1.5%) referred persistent local pain after 12 months follow-up and inability to return to professional sport activity.

In our series, return to full sport activity was 94.4% (187 patients) at 4 weeks and 98.5% (195 patients) at 12 months. Only 3 patients (1.5%) were unable to return to elite sport activity because of non-responsive chronic inguinal pain.

One patient (0.5%) returned to our attention after 45 months from surgery referring monolateral pain; both clinical examination and inguinal US were negative for disease recurrence. The patient was treated conservatively with sport activity pause for 3 months with complete symptom remission and full return to professional sport activity.

Furthermore, in a long-term follow-up, we reported 5 cases (2.5%) of ID recurrence in previously asymptomatic patients: 1 patient (0.5%) after 13 months was treated with conservative therapy; 2 patients (1.0%) after 23 and 29 months were treated with open surgical approach; 1 patient (0.5%) after 48 months was treated with open surgical approach; 1 patient (0.5%) after 60 months was not submitted to surgical treatment because of the patient’s will to stop professional sport activity. All patients with recurrence had inguinal monolateral pain associated to a small groin tumescence. All patients referred recurrence after full return to professional sport activity. At clinical examination, monolateral hernia was diagnosed. Surgical treatment with an open approach (Lichtenstein technique) was performed in 3 patients. Return to full sport activity was possible after 13 weeks from surgery.

## Discussion

**Fig. 6:**
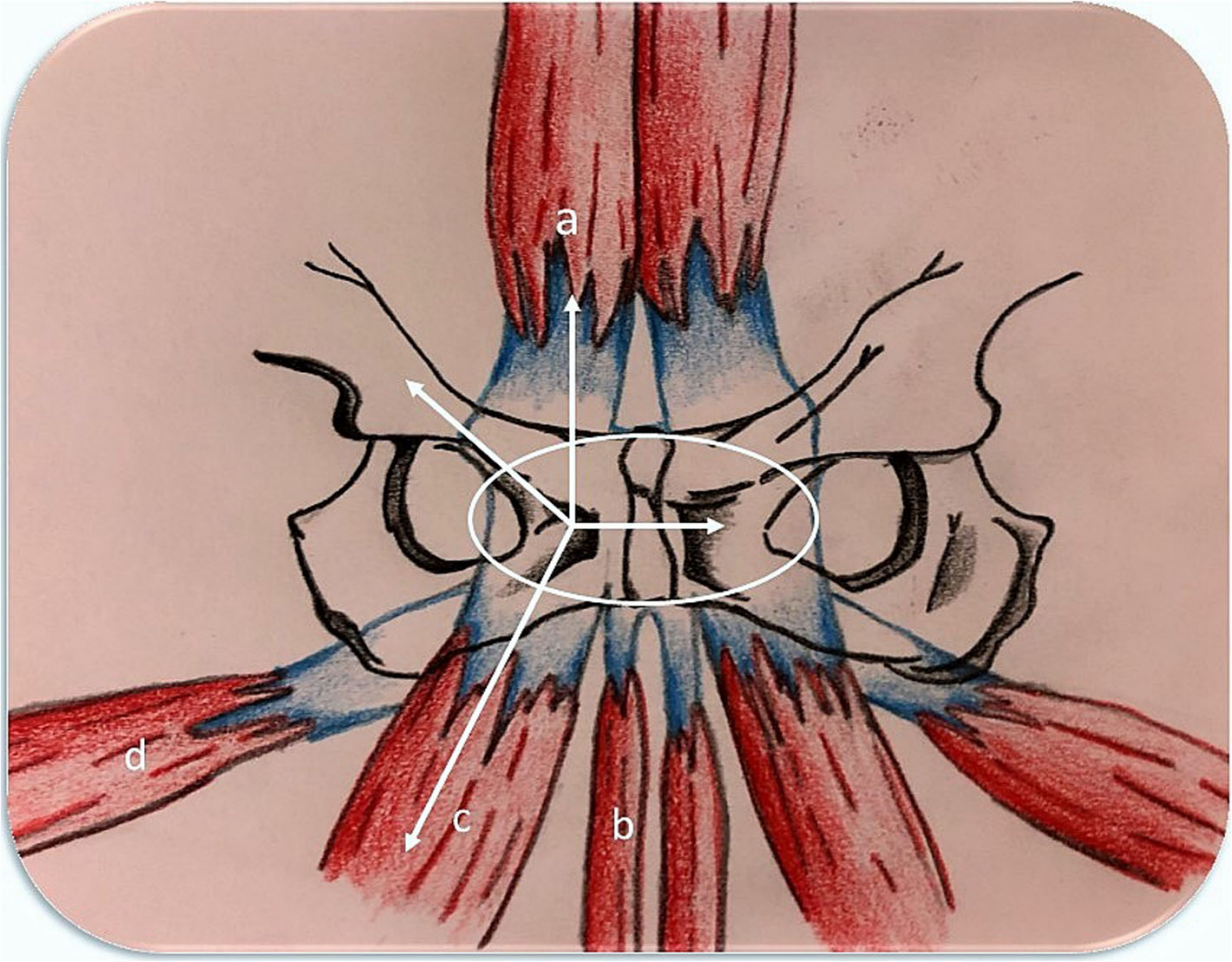
Scheme of the musculotendinous and aponeurotic attachments of the anterior pubis. Ellipse—prepubic aponeurotic complex (P-PAC); a—rectus abdominis, b—gracilis, c—adductor longus, d—adductor brevis. The arrows schematize the shearing forces with P-PAC as fulcrum

The Manchester Consensus Conference of the British Hernia Society (BHS) [4] aimed to pinpoint a “blurry” condition characterized by chronic groin pain often evidenced in elite athletes. This consensus managed to black out all the previous unspecific terms agreeing in the use of a more specific “inguinal disruption” [4]. A real hernia is often unseen therefore making the term “hernia” not adequate for this chronic condition. Instead, we believe that the term ID accurately describes the tissue distress in the inguinal area following an important increase in local tension due to the high level of “twisting, turning, sprinting and kicking” that the athletes undertake during intense sport activity [4]. Consequently, these sportsmen have a disequilibrium in forces applying in the inguinal area, which is composed of unextendible structures (e.g. pubic bone, inguinal ligament) and relatively extendible structures (e.g. external inguinal ring, conjoint tendon, external oblique aponeurosis) in tight relation, with a following high risk of tissue tearing. In particular, the anterior aspect of the symphysis pubis is composed of a soft-tissue complex known as prepubic aponeurotic complex (P-PAC). P-PAC is composed of interconnections between the adductor tendons, rectus abdominis, inguinal musculo-aponeurotic structures, articular disc and pubic ligaments of the symphysis pubis [15] (Fig. 6). ID is therefore a complex condition that overlaps other clinical conditions (e.g. adductor muscle tendinitis, osteitis pubis or pubic symphysitis) although it is accepted that they can coexist [16, 17]. BHS proposed interesting 5-point diagnostic clinical criteria for ID (Table 2) [4]; however, clinical evidence is needed for validation. BHS also suggested a management algorithm for ID (Table 3). Specific rehabilitation programs and physiotherapy with associated rest and analgesia are strongly recommended for all athletes in the first instance [4, 18]. However, the BHS states that further discussion is needed about the conservative treatment options [4]. Patients should be submitted to at least 2 months of conservative treatment (Table 3); however, as supported by the authors, this therapeutic protocol is often considered inadequate for elite professional athletes’ demands of a fast recovery. Caudill et al. performed a systematic literature review analysing a total of 23 published surgical series for sports hernia further highlighting the role of surgery as a definitive treatment. A success rate of 63–97% is reported for both symptom relief and return to previous sport activity associated to surgical treatment (open or laparoscopic). In the same study also, comparable results are reported between open (92.8 ± 9.9%) and laparoscopic (96.0 ± 4.5%) repair based only on the criterion of return to sport activity [16].

Furthermore, Ekstrand et al. showed, in a randomized controlled trial (RCT) including 66 soccer players, that only surgical treatment (open approach) was associated to significant symptom improvement compared to conservative treatment (individual training, anti-inflammatory, analgesics and physical therapy) [19]. Additionally, Paajanen et al. reported that conservative treatment is less effective in reducing pain compared to surgery (TEP approach) with the latter associated to excellent immediate and long-term relief of pain [20].

This study supports the results of the two RCT that are mentioned above [19, 20], and in our opinion, laparoscopic surgery is the mainstay for non-responsive ID treatment. All the 198 patients of our series underwent unsuccessful conservative treatment with anti-inflammatory drugs, rest and physiotherapy for at least 6 weeks. The patients were athletes from various sports (Table 4) especially related to “twisting, turning, sprinting and kicking” movements as previously discussed. 92.4% of our patients were submitted preoperatively to dynamic abdominal wall ultrasound (dUS) with evidence of peritoneal fat ballooning during Valsalva manoeuvre in 88.4% of patients. MRI was performed in 8 patients (4.0%) in order to confirm an inguinal bulge not detected under US. The goal of imaging in ID is to accurately evaluate the muscular-aponeurotic structures of the inguinal area. MRI is considered the best tool (sensitivity 98%; specificity 89-100%) in patients with ID for injuries involving the rectus abdominis, the adductor tendon origin and the symphysis itself [21]. MRI is also useful in evaluating the regional bone structures as possible sites of associated osseous stress structures [21]. Despite the aforementioned role of MRI as a primary diagnostic tool, we decided to perform it only if a dUS was not fully diagnostic. In fact, we use dUS as a primary diagnostic tool in association to clinical evaluation for 2 reasons: primarily, because the dUS was always performed by a skilled radiologist with long-term experience on inguinal evaluation, and secondarily, because of the lower cost of dUS compared to MRI. Therefore, MRI was performed only in clinical doubt (8 patients in our series).

The primary aim in ID is to improve the symptoms and enable a fast return to sport activity. Surgery, regardless of open or laparoscopic approach, is considered quite effective for fast return to sport activity [16]. However, time for return to full sport activity is different between laparoscopic and open repair. Srinivasan et al. reported in a small series of 15 patients treated laparoscopically a rate of 87% of training starting at 4 weeks after surgery and return to full activity within 6 weeks with no recurrent symptoms at 12.1 months (range 6–60 months) [22]. Ingoldby reported that 13 of 14 athletes (92.9%), who underwent laparoscopic surgery for ID, returned to training in 4 weeks [23]. Open repair is instead generally associated to return to full activity at 6 months post-surgery as reported by Ahumada et al. in a case series of 12 patients [24], at 14 weeks post-surgery as reported by Kumar et al. in a case series of 27 patients [25] and at 6 months post-surgery as reported by Malycha et al. in a case series of 44 patients [26].

Furthermore, patients submitted to open approach require to be relatively inactive for the initial four postoperative weeks [24]. Therefore, both open and laparoscopic approaches are effective for ID treatment; however, laparoscopy offers the advantage of a faster rehabilitation, an earlier return to unrestricted activities of daily living and earlier return to full sport activity. According to our results, in a series of 198 patients, 187 patients (94.4%) started full sport activity at 4 weeks with a total of 195 patients (98.5%) active at 9 months. Our results are compliant to literature results [20, 22, 23], showing furthermore the advantage of laparoscopy, in our case series with the TAPP technique, over open approach. Moreover, we believe that the TAPP technique is superior to open approach because it permits to explore the abdominal cavity in order to exclude concurrent clinical entities (such as adherences from previous surgeries), and it is associated to a reduced risk of post operatory neuralgia related to nerve sparing surgery. However, no significant difference has been described between TAPP and TEP regarding operative time and post-operative neuralgia [27].

Although the surgical site depends on the reported symptoms and diagnosis of ID [4, 16, 22], the authors believe that TAPP repair should be performed bilaterally in ID even if the symptoms are monolateral in order to have a better outcome. As previously described, athletes are constantly submitted to “twisting, turning, sprinting and kicking” actions that may result in local tears of the P-PAC with consequent disequilibrium of forces applying in the inguinal area and disruption of the inguinal-pubic region. Therefore, performing a TAPP procedure monolaterally could not fully balance and stabilize the anatomical region. Also, in elite professional athletes, the treatment must be radical in order to return sooner to full sport activity with no risk of contralateral occurrence of symptoms. Further studies are necessary to demonstrate the relationship between bilateral repair and the clinical outcome.

Post-surgical inguinal pain is the most frequent complication associated to inguinal hernia repair. Several improvements in anatomic knowledge, adoption of “tension free” techniques and the use of lightweight mesh are associated to reduced post-surgical pain [28]. In particular, Li et al. reported in a metanalysis of 5389 patients that the lightweight mesh repair was associated to a significant minor incidence of chronic postoperative pain [OR = 0.72, 95% CI (0.57, 0.91)] and to a reduced feeling of foreign body compared to heavyweight mesh repair [OR = 0.50, 95% CI (0.37, 0.67)] regardless of the surgical approach [28]. In our case series, we performed hernia repair using a lightweight tailored non-absorbable polypropylene monofilament mesh (BULEV B®; 48 g/m2 ± 10%). Furthermore, Sajid et al. demonstrated in a meta-analysis of 1001 patients the reduced risk for developing chronic groin pain associated to glue mesh fixation, compared to tacker mesh fixation (RR 4.64; 95% CI, 1.85–11.66, *P* < 0.001), [29]. We performed both the fixation of the mesh and the closure of the peritoneal flap with n-hexyl/cyanoacrylate glue (IFABOND®).

In this study, the main complication was post-surgical pain as described in literature. At 4 weeks, 11 patients (5.6%) referred chronic inguinal pain with delayed return to sport activity. These 11 patients were submitted to medical treatment and physiotherapy. Chronic inguinal pain resolution and return to sport activity was reported by 5 patients after 3 months, 1 after 6 months and 2 after 9 months. Only 3 patients (1.5%) were unable to return to elite sport activity because of non-responsive chronic inguinal pain. In addition, in a long-term follow-up of 13 years, we reported a total of 5 cases (2.5%) of ID recurrence in previously asymptomatic patients.

Since this is a retrospective study, it was not possible to characterize the temporal dynamics of postoperative pain intensity and this could be considered a limitation of this study. All the pain symptoms were retrieved by analysing past clinical reports and contacting patients at the time of the study. Furthermore, the first 15 patients of this series were not submitted to a preoperative dUS due to a not fully standardized diagnostic and treatment protocol for ID. This could be considered another possible limitation of this study.

Despite the fact of being retrospective, this study could give important details further confirming previously published data in literature. This study evaluates a wide surgical period (13 years) with a long-term follow-up. Moreover, all cases have been highly standardized by performing high-quality ultrasound preoperative evaluation and a standardized surgical procedure in a single surgeon setting.

To our knowledge, this series is the widest described single operator TAPP experience in ID in literature. We further demonstrate the efficacy and safety of TAPP technique in ID treatment.

## Conclusion

ID is a chronic groin pain affecting mainly athletes characterized a multifactorial pathogenesis. Diagnosis of ID is a clinical challenge, and it is frequently associated to secondary or tertiary clinical conditions. Clinical evaluation is fundamental for diagnosis; however, dUS and MRI are useful for achieving a precise diagnosis. The British Hernia Society has proposed diagnostic criteria and a therapeutical algorithm in which surgery is the treatment of choice when conservative treatment fails. Surgical treatment can be either open or laparoscopic. The TAPP approach is safe and effective for the treatment of ID, and it is associated to a faster return to full sport activity. Chronic groin pain is the most frequent post-surgical complication, and it affects a full recovery with delay of sport activity. Our case series is compliant to literature results. To our knowledge, our series is the widest described single operator TAPP experience in ID in literature.

## Data Availability

The datasets used and/or analysed during the current study are available from the corresponding author on reasonable request.

## Abbreviations

BHS: British Hernia Society
dUS: Dynamic abdominal wall ultrasound
ID: Inguinal disruption
LT: Laparoscopic technique
MRI: Magnetic resonance imaging
OT: Open technique
PIPS: Pubic inguinal pain syndrome
POD: Post-operative day
P-PAC: Prepubic aponeurotic complex
RCT: Randomized controlled trial
TAPP: Transabdominal preperitoneal patch plasty
TEP: Total extraperitoneal patch plasty
US: Ultrasound
